# Aerobic training improves exercise capacity after traumatic brain injury in female, but not male, mice

**DOI:** 10.3389/fphys.2025.1700462

**Published:** 2025-10-30

**Authors:** Kate Karelina, Deborah Corbin, Claymore T. Gumbo, Taylor Payne, Emma Reger, Jayden Barr, Mikayla Oldham, Brett Shoemaker, Sakthijothi Muthu, Ethan Meadows, John M. Hollander, Zachary M. Weil

**Affiliations:** ^1^ Department of Neuroscience, Rockefeller Neuroscience Institute, West Virginia University, Morgantown, WV, United States; ^2^ Department of Physiology, Pharmacology and Toxicology, West Virginia University, Morgantown, WV, United States; ^3^ Department of Human Performance – Exercise Physiology, West Virginia University, Morgantown, WV, United States

**Keywords:** traumatic brain injury, aerobic exercise, exercise intolerance, cardiovascular fitness, sex differences

## Abstract

**Introduction:**

Traumatic brain injury (TBI) often leads to lasting impairments in physical performance, yet its impact on aerobic function and the potential for recovery through exercise remain poorly understood. In this study, we used a well-established controlled cortical impact (CCI) model in mice to address three gaps in the preclinical TBI literature: the effect of injury on voluntary activity and energy metabolism, the extent to which exercise tolerance and cardiorespiratory fitness can be restored through moderate-intensity aerobic training, and whether these responses differ between sexes.

**Methods:**

Voluntary wheel running and metabolic outputs following CCI were quantified via the Comprehensive Lab Animal Monitoring System (CLAMS), while maximal oxygen uptake (VO_2_ max) and time to exhaustion were measured before and after a 10-day treadmill training regimen initiated during the subacute phase.

**Results:**

Both sexes displayed similar acute reductions in VO_2_ max following TBI; however, only females exhibited significant gains in VO_2_ max and exercise tolerance after exercise training, alongside higher spontaneous activity, greater energy expenditure, and smaller lesion volumes compared to males. Exercised females also exhibited selective cardiac upregulation of mitochondrial complex activity, indicating that enhanced mitochondrial capacity paralleled improved aerobic performance. In contrast, injured males showed persistent deficits and no measurable improvement in cardiovascular fitness or mitochondrial physiology from training, indicating a sex-specific limitation in aerobic adaptation.

**Discussion:**

These findings reveal a divergence in post-TBI exercise responsiveness and highlight the need for sex-specific, physiology-guided rehabilitation strategies.

## Introduction

Traumatic brain injury (TBI) remains a leading cause of long-term disability worldwide, resulting in persistent cognitive, emotional, and physical impairments (Arciniegas and Wortzel, 2014; Bramlett and Dietrich, 2015; Stocchetti and Zanier, 2016). Among these, reduced exercise capacity is an underrecognized but clinically significant consequence that may hinder recovery and limit the effectiveness of rehabilitation (Antonellis et al., 2024; Hamel and Smoliga, 2019; Mossberg et al., 2007). In individuals with moderate to severe TBI, maximal aerobic capacity can fall to the 5^th^ percentile, and cardiovascular dysfunction may persist for years (Hamel and Smoliga, 2019). Therefore, although aerobic exercise is well established as a therapeutic strategy for improving cardiovascular and neurological health, its utility as a rehabilitation approach after TBI may be constrained by a reduced capacity to engage in exercise.

Clinical studies suggest that TBI impairs both exercise capacity, defined as the maximal physical performance an individual can achieve (e.g., VO_2_ max) (Rowe et al., 2023), as well as exercise tolerance, defined as the ability to sustain activity without premature fatigue or symptom-limited cessation (Ekkekakis et al., 2005). These deficits likely arise from a combination of central and peripheral dysfunctions, including autonomic dysregulation, impaired myocardial performance, altered baroreflex sensitivity, and reduced cerebral perfusion during exertion (Ellingson et al., 2022; Esterov and Greenwald, 2017; Tan et al., 2014). Together, these impairments may limit both oxygen delivery and metabolic flexibility during physical activity, ultimately diminishing the effectiveness of exercise-based interventions. Further, cardiometabolic deficits after TBI could potentially yield deleterious neurological and functional consequences from high-intensity training. Moreover, given the high energetic demands of the heart and the known plasticity of the cardiovascular system following both injury and training (Kohlhaas et al., 2017) we hypothesized that mitochondrial bioenergetics may be a critical mediator of exercise capacity after TBI. Mitochondria regulate cardiac output and shift metabolic fuel preference, both of which can be remodeled by endurance training (Heinonen, 2025; Leone and Kelly, 2011). However, it remains unspecified whether similar metabolic adaptations occur in the heart after TBI, or whether they contribute to sex differences in recovery. Although aerobic training improves exercise tolerance and cardiovascular efficiency in a range of disease states (Araujo et al., 2023; Farid et al., 2005; Jeppesen et al., 2006; Rampello et al., 2007), no preclinical studies to date have directly evaluated whether aerobic training can restore cardiovascular fitness or exercise tolerance after TBI, or whether such adaptations are supported by mitochondrial reprogramming.

Compounding this gap is a persistent lack of attention to sex as a biological variable in both clinical and preclinical TBI research. Males and females differ markedly in their baseline physiological responses to exercise, including voluntary activity levels, substrate utilization, and cardiovascular regulation; differences shaped by sex hormones, body composition, and metabolic efficiency (Ansdell et al., 2020; Bassareo and Crisafulli, 2020; Cano et al., 2022; Hunter et al., 2023; Rosenfeld, 2017). In parallel, established sex-specific vulnerabilities to TBI pathophysiology, such as differences in lesion volume, neuroinflammation, and axonal degeneration, are thought to be mediated, in part, by hormonal modulation of inflammatory and cell death pathways (Gupte et al., 2019; Haynes and Goodwin, 2023). Together, these factors suggest that sex may strongly influence both the capacity to engage in and respond to aerobic rehabilitation following TBI. Yet few studies have evaluated whether these sex differences translate into divergent responses to post-TBI aerobic training, particularly with respect to exercise tolerance and cardiovascular recovery.

Previously our lab has shown that there are prominent sex differences in the beneficial metabolic, neuropathological, and functional consequences of graded treadmill training after TBI (White et al., 2023). Specifically, we have reported that injured male mice that run at low or moderate speeds exhibit reduced cortical lesion volume and improved spatial learning and memory. However, high intensity exercise regimens impaired both cognitive and mitochondrial function. In contrast, injured females benefitted from all exercise intensities, albeit to a lesser extent. However, it remains unspecified whether brain injury altered exercise capacity and thus differentially affected responses to treadmill training.

In this study, we use a well-characterized mouse model of controlled cortical impact (CCI) to address three major gaps in the TBI literature. First, we assess how TBI affects voluntary exercise behavior and associated metabolic outputs using indirect calorimetry and wheel-running activity. Second, we evaluate how TBI impacts exercise tolerance, cardiorespiratory fitness, and cardiac metabolic function to determine whether these measures can be improved through moderate-intensity aerobic training initiated during the subacute recovery phase. Finally, we determine the extent to which these outcomes differ by biological sex.

## Methods

### Animals

All procedures were conducted on male and female Swiss Webster mice (∼6 weeks of age at the time of injury) derived from breeders purchased from Charles River (Wilmington, PA). Pups were weaned at 21 days of age and housed in a standard mouse cage with *ad libitum* access to food and filtered tap water. All procedures were approved by the Institutional Animal Care and Use Committee at West Virginia University. The treatment of animal subjects was in accordance with the ethical standards of the NIH, and the authors complied with ARRIVE 2.0 guidelines. All animals were randomly assigned to experimental conditions and investigators were blinded to group assignments during data collection and analysis.

**FIGURE 1:**
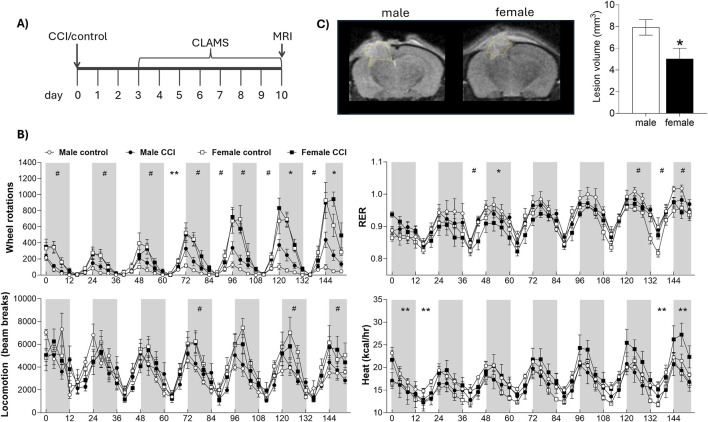
Sex differences in lesion volume and indirect calorimetry following CCI. **(A)** Mice underwent CCI or control surgery and were then housed in metabolic CLAMS cages for 7 days, followed by MRI scanning. **(B)** CLAMS data are shown over 7 days. Gray boxes represent the dark phase; white areas represent light phase. Metabolic phenotyping revealed effects of sex and surgery on respiratory exchange ratio (RER), wheel rotations, heat production, and total locomotion*.* **(C)** Lesion volume was significantly smaller in female mice compared to males. # indicates significant effect of sex; * indicates significant effect of surgery; ** indicates significant sex by injury interaction. Data were considered statistically significant at p < 0.05.

**FIGURE 2:**
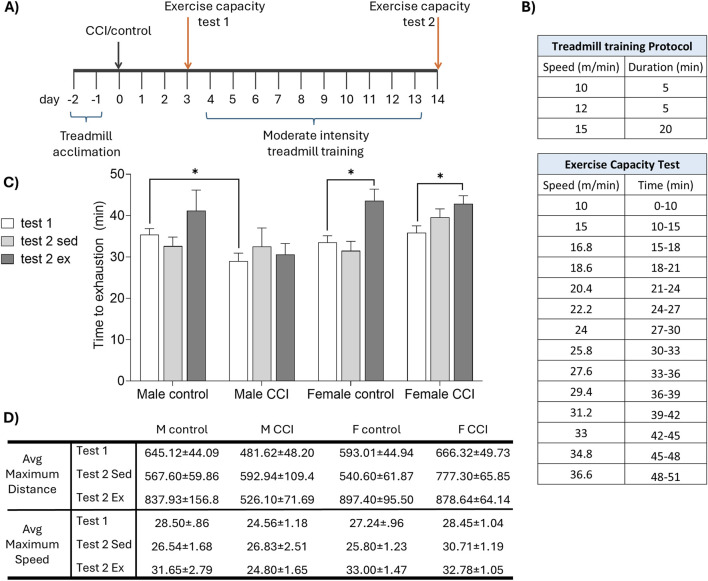
Sex differences in recovery of exercise capacity following CCI. **(A)** Mice were acclimated to the treadmill, then underwent CCI or control surgery. The exercise capacity was assessed before (test 1) and after (test 2) 10 days of moderate intensity treadmill training (or sedentary control). **(B)** Speed and duration of (top) the daily treadmill training protocol and (bottom) exercise capacity test. **(C)** Time to exhaustion on the treadmill fatigue test. CCI acutely reduced exercise capacity in male, but not female, mice. Exercise training significantly improved exercise capacity in female but not male CCI mice. **(D)** Greatest distance (in meters) and speed (meters per minute) achieved by each group. Data in tables are presented as mean (±SEM). *indicates significant difference (p < 0.05) between the indicated groups.

**FIGURE 3:**
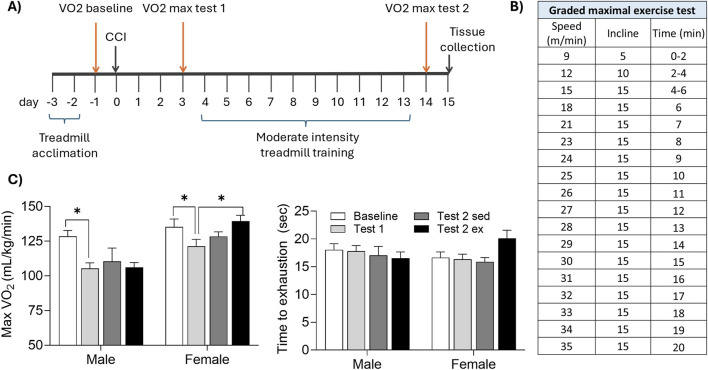
Aerobic exercise enhances maximal oxygen uptake in injured female, but not male, mice. **(A)** Mice were acclimated to the treadmill, then underwent CCI surgery. A graded maximal exercise test was conducted 1 day before CCI (baseline), 3 days after CCI (test1), and again following 10 days of moderate intensity treadmill training (or sedentary control). **(B)** Speed, incline, and duration of the graded maximal exercise test. **(C)** CCI acutely reduced maximal oxygen uptake (max VO_2_) in male, but not female, mice. Moderate intensity exercise significantly increased max VO_2_ in female but not male mice. *indicates significant difference (p < 0.05) between the indicated groups.

Three cohorts of mice were tested. Mice in cohort 1 underwent controlled cortical impact (CCI) or control surgery. Beginning on the 3^rd^ day of recovery, mice were individually housed in CLAMS metabolic cages for 7 days, followed by MRI lesion volume assessment (Figure 1A; control females n = 5, CCI females n = 8, control males n = 5, CCI males n = 8). In cohort 2, CCI and control mice underwent an exercise capacity test before and after a 10-day treadmill training protocol (Figure 2A; control sedentary females n = 6, control exercised females n = 4, CCI sedentary females n = 8, CCI exercised females n = 8, control sedentary males n = 6, control exercised males n = 4, CCI sedentary males n = 7, CCI exercised males n = 9). In cohort 3, CCI mice were assessed for aerobic capacity in a graded maximal exercise test before and after a 10-day treadmill training protocol (Figure 3A; sedentary females n = 5, exercised females n = 7, sedentary males n = 5, exercised males n = 6).

### Controlled cortical impact

Traumatic brain injury was induced using the CCI model as previously described (White et al., 2023). Briefly, mice were anesthetized with inhaled isoflurane (3% in oxygen) and secured into a stereotaxic frame equipped with a heating pad (Stoelting). Their skulls were then exposed and a 4 mm craniectomy was performed over the left parietal bone. A 3 mm plunger was retracted and accelerated into the brain inducing a 1.2 mm deformation at a velocity of 5 m/s, with a dwell time of 30 msec. The control group had their skulls exposed but no craniectomy or injury was conducted. After CCI or control surgery the incision was sutured with 6/0 nylon suture and mice were replaced into their home cages. All mice were treated subcutaneously with a cocktail of bupivacaine/lidocaine (1.5 and 0.5 mg/kg respectively) and meloxicam (5 mg/kg) for analgesia and monitored daily post-operatively. A total of 6 mice died within 24 h of surgery, 3 mice were euthanized while still under anesthesia due to development of a skull fracture during the CCI procedure, and 1 mouse was euthanized for excessive weight loss. Following injury, mice were checked regularly for grooming, posture, spontaneous locomotion, hydration, and surgical site appearance.

### Treadmill training

Mice were acclimated to the enclosed treadmill (Columbus Instruments Exer 3/6) over 2 days (day 1: 8 m/min for 5 min, 9 m/min for 2 min, 10 m/min for 3 min; day 2: 10 m/min for 5 min, 11 m/min for 5 min, 12 m/min for 5 min) (Dougherty et al., 2016). Moderate intensity treadmill training was conducted over 10 days as previously reported (10 m/min for 5 min, 12 m/min for 5 min, 15 m/min for 20 min) at a 0° incline, with no aversive shock stimuli (White et al., 2023). During treadmill training, mice were observed for gross motor ability, measured as sustained ambulation at all speeds; no animals were excluded for inability to run. Mice in sedentary conditions were placed on the treadmill for 30 min, with the treadmill off. The treadmill was cleaned with 70% ethanol between mice.

### Exercise capacity testing

Exercise capacity testing, adapted from Dougherty et al. (2016), was conducted 3 days after CCI or control injury (test 1) and again following the completion of the treadmill training protocol (test 2). Mice were acclimated to the enclosed treadmill as described above. For exercise capacity testing, mice were placed on the treadmill set to a 10° incline and the treadmill speed was progressively increased (see Figure 2). Exercise capacity was defined as the time at which a mouse remained within one body length of the back of the treadmill for 10 consecutive seconds. Total distance and maximum speed achieved were also recorded. Shock stimuli were not used, and the treadmill was cleaned with 70% ethanol between mice.

### CLAMS indirect calorimetry

Beginning on the third day of recovery from CCI or control surgery, mice were individually housed in the Comprehensive Lab Animal Monitoring System (Oxymax-CLAMS; Columbus Instruments). Respiratory parameters (oxygen consumption, VO_2_ and carbon dioxide production, VCO_2_), locomotion (Y and X-axis infrared beam breaks), wheel rotations, and food intake were collected over 7 days. Respiratory exchange ratio (RER: VCO_2_/VO_2_) and energy expenditure (“heat”: kcal/hr normalized to body mass) were calculated from respiratory parameters. Data were analyzed in 4-h bins by a blinded observer. For all CLAMS analyses, the first 6 h were excluded to allow gas concentrations within the chambers to stabilize following the transition from ambient air. Time 0 is defined as the first data point after this 6-h stabilization period and coincides with the onset of the first dark cycle.

### Graded maximal exercise test

Mice were acclimated to treadmill running as described above. Aerobic capacity was measured using the CLAMS metabolic modular treadmill protocol at three timepoints; 1 day before CCI (baseline), 3 days after CCI (test 1), and again following the completion of the treadmill training protocol as described above (test 2). Parameters for measuring gas exchange were set as described by Petrosino et al. (2016). Each graded maximal exercise test was conducted on a gradually increasing incline (5°–15°) with a progressive increase in speed until exhaustion (see Figure 3), defined as continuous contact with the shock grid (1.5 mA) for five consecutive seconds. Respiratory parameters (VO_2_ and VCO_2_) were collected throughout the test. Maximal oxygen uptake (VO_2_ max) was identified using CLAX software by a blinded observer.

### MRI

Mice were anesthetized with 1.5%–2% isoflurane and positioned into a head coil (23 mm) in an Aspect M7 MRI scanner (Aspect Imaging). Body temperature was maintained at 37 °C using a heated water circulator pump, and respiration was monitored via a respiratory cushion. T2 weighted images were acquired with the following parameters: field strength = 1.05 T, repetition time (TR) = 3s, echo time (TE) = 66 m, FOV = 30 mm × 30 mm, matrix size = 192 × 192, slice thickness = 1 mm, no interslice gap, and 9 min 15s imaging time. MRI DICOM files were analyzed for lesion volume using VivoQuant™ software via manual delineation of the lesion based on hyperintensity in T2-weighted images. Lesion tracing and data analysis were conducted by a blinded observer.

### Electron transport chain (ETC) complex activity

Mice were euthanized via decapitation, and hearts were harvested, snap-frozen, and stored at −80 °C until further analysis. Cardiac mitochondria were isolated (Croston et al., 2013), and electron chain complex (ETC.) I-V activities were measured as previously described (Geldenhuys et al., 2019; Silva et al., 2009). Briefly, complex I activity was measured in 5 µg mitochondria per well following the addition of 1 µM rotenone to the buffer (50 mM phosphate (pH 7.4), 2 mM KCN, 5 mM MgCl_2_, 2.5 mg/mL BSA, 2 µM antimycin-A, 100 µM decylubiquinone, and 0.3 mM NADH). Absorbance was measured at 340 nm. Complex II activity was measured in 5 µg mitochondria per well following the addition of 0.5 mM TTFA to the buffer (25 mM phosphate (pH 7.4), 2 mM KCN, 20 mM succinate, 50 µM DCIP, rotenone 2 μg/mL, antimycin-A 2 μg/mL, and 56 µM decylubiquinone). Absorbance was measured at 600 nm. Complex III activity was measured in 2.5 µg mitochondria per well following the addition of 2 µM antimycin to the buffer (25 mM phosphate (pH 7.4), 1 mM EDTA, 1 mM KCN, 0.6 mM dodecyl maltoside, 75 µM oxidized cytochrome c, and 100 µM decylubiquinol). Absorbance was measured at 550 nm. Complex IV activity was measured in 1.25 µg mitochondria per well in a Tris-HCl, KCl and KCN buffer with a 1:20 ratio of reduced cytochrome c solution. Absorbance was measured at 550 nm. Complex V was measured in 2.5 µg mitochondria per well following the addition of 20 µL ATP to the working buffer (HEPES/KCl/Mg^2+^/Pi/PEP/KCN/glycerol) containing 200 µM NADH and PK/LDH coupling enzymes). Absorbance was measured at 340 nm. All samples were run in duplicate, included appropriate negative controls, and were analyzed on a Flexstation 3 plate reader after a 5-min incubation.

### Statistics

Statistical comparisons for CLAMS indirect calorimetry data were performed separately for each 12-h light (inactive) and dark (active) cycle using two-way ANOVA (sex x injury). Lesion volume was assessed using a one-way ANOVA (sex). One-way ANOVA (sex) and paired t-tests (baseline vs. test 1, and test 1 vs. test 2) were used for data comparison from the exercise capacity and graded maximal exercise test results. Cardiac mitochondrial complex activity was assessed via ANOVA and independent t-tests. Results were considered significant for p ≤ 0.05. Statistical analyses were conducted using SPSS v. 29, graphs were produced using GraphPad Prism 10.

## Results

### Voluntary wheel running and indirect calorimetry

To assess the impact of CCI on metabolic function, control and injured mice were housed individually in CLAMS metabolic cages equipped with running wheels for a continuous 7-day period, beginning on post injury day 3 (Figure 1A). Both control and CCI female mice exhibited a marked increase in voluntary wheel running, particularly toward the end of the analysis period, spending two-to four-fold more time on the wheels than males at all active time points (e.g., 120–132 h: F_1,21_ = 43.59, p < 0.001, male vs. female). There was no significant effect of injury of wheel running in female mice (all p > 0.05, control vs. CCI in females). Further, while CCI males showed a modest increase in wheel activity on nights 6 and 7 compared to control males, this was substantially less than the wheel running observed in females (e.g., 120–132 h: F_1, 11_ = 5.596, p < 0.05, control vs. CCI in males). Locomotor activity, as measured by X- and Y-axis beam breaks along the cage floor, revealed only subtle sex differences that reached significance on select nights (e.g., 72–84 h: F_1,21_ = 4.318 p = 0.05, male vs. female), but no significant effect of injury (all p > 0.05; control vs. CCI), indicating that the enhanced exercise capacity in injured females was not associated with a more generalized hyperactivity. Overall, CCI had minimal effect on the RER, however, female mice exhibited significantly higher RER values, particularly on days 6 and 7 (e.g., 120–132 h: F_1,22_ = 6.952, p < 0.05, male vs. female; Figure 1B). Energy expenditure, measured as heat production, varied significantly over time with a notable sex × injury interaction. Specifically, male control mice exhibited higher energy expenditure early in the testing period (e.g., 12–24 h: F_1,20_ = 2.325, p < 0.05, surgery × sex interaction), whereas female CCI mice showed a marked increase in energy expenditure during the final 24 h (e.g., 144–152 h: F_1,20_ = 4.25, p = 0.05, surgery × sex interaction; Figure 1B). Cumulative food intake was significantly greater in males (F_1,22_ = 10.783, p < 0.01, male vs. female), but did not differ between control and CCI groups (p > 0.05; Supplementary Figure S1). Lesion volume, assessed via MRI immediately following the CLAMS monitoring period, revealed a significant sex difference, with females exhibiting significantly smaller lesion volumes compared to males (F_1,13_ = 7.894, p < 0.05; Figure 1C); however, lesion volume did not significantly correlate with any metabolic measures obtained from CLAMS monitoring (all p > 0.05; data not shown).

### Exercise capacity

To further evaluate the impact of CCI on exercise capacity, a separate cohort of control and CCI male and female mice was subjected to a treadmill exhaustion protocol 3 days after injury and again following a 10-day period of moderate intensity treadmill training or sedentary control conditions (Figures 2A,B). All mice exhibited normal locomotion after surgery and were able to complete the exercise capacity task. CCI acutely impaired exercise capacity in males, evidenced by a significant reduction in time to exhaustion compared to controls (F_1,24_ = 5.46, p < 0.05 test 1 control vs. CCI males). In contrast, exercise capacity in injured female mice remained comparable to uninjured controls (F_1,24_ = 0.005, p > 0.05 test 1 control vs. CCI female). Following 10 days of moderate intensity treadmill training, time to exhaustion was significantly increased in both control (*t* (3) = 3.661, p < 0.05) and CCI females (*t* (7) = 4.706, p < 0.05) but failed to increase in males (Figure 2C). Maximum running distance and peak speed achieved during the exercise capacity test followed patterns consistent with time to exhaustion (Figure 2D).

## Aerobic capacity during a graded maximal exercise test

Aerobic capacity, defined as maximal oxygen consumption (VO_2_ max) was assessed during the graded maximal exercise test (Figures 3A,B). At baseline testing, both sexes reached similar VO_2_ max values (p > 0.05 male vs. female). Both sexes demonstrated a significant reduction in VO_2_ max acutely following CCI (*t* (22) = 3.836, p < 0.05 baseline vs. test 1). However, VO_2_ max significantly improved after moderate intensity treadmill training in females (*t* (6) = 4.221, p < 0.05 test 1 vs. test 2), but not males (p > 0.05).

### Electron transport chain complex activity

Mice were euthanized within 24 h of completing the graded maximal exercise test and hearts were harvested for electron transport chain complex activity (Table 1). Among the five ETC, complexes analyzed, complex I activity was significantly increased by sex (F_1,32_ = 4.088, p = 0.05), exercise (F_1,32_ = 4.848, p < 0.05), and injury (F_1,32_ = 5.049, p < 0.05); complex III (F_1,32_ = 5.535, p < 0.05) and complex IV (F_1,32_ = 4.488, p < 0.05) activities were both significantly increased by injury, and complex V activity was significantly greater in females compared to males (F_1,32_ = 37.302, p < 0.01). Independent t tests confirmed that complex I (*t* (9) = 2.525, p < 0.05) and complex III (*t* (9) = 2.233, p = 0.05) activity was significantly increased by exercise in injured females compared to controls; and complex IV activity (*t* (8) = 2.361, p < 0.05) was significantly increased by exercise in injured males compared to controls.

**TABLE 1:** Exercise enhances cardiac mitochondrial complex I and III activity in injured female mice, and complex IV activity in injured males.

Electron transport chain complex activity					
Group	Complex I^*#†^	Complex II	Complex III^†^	Complex IV^†^	Complex V^*^
Male Control	407.26 (42.4)	537.96 (25.9)	509.46 (72.2)	632.43 (90.7)	370.97 (23.7)
Male CCI + Sed	482.26 (40.9)	618.46 (49.7)	778.46 (114.2)	847.65 (127.2)	564.56 (113.6)
Male CCI + Ex	512.18 (46.3)	583.33 (24.4)	616.22 (58.5)	846.84 (44.5)**	473.12 (43.1)
Female Control	411.29 (26.3)	520.29 (23.1)	524.32 (61.6)	627.03 (109.7)	806.45 (76.2)
Female CCI + Sed	589.72 (94.6)	523.23 (34.9)	629.73 (65.3)	727.03 (95.4)	913.31 (92.6)
Female CCI + Ex	795.05 (108.7)**	553.93 (40.0)	736.80 (62.1)**	883.09 (89.4)	1,020.92 (98.4)

Complex activity was significantly altered by sex, exercise, and injury. * indicates significant effect of sex; # indicates significant effect of exercise; † indicates significant effect of CCI; ** indicates significantly different from control. Activities of electron transport chain (ETC) complexes I-V are shown as mean nmol/min/mg (±1 SEM).

## Discussion

Here, we demonstrate that moderate-intensity aerobic training improves exercise capacity and aerobic fitness following traumatic brain injury (TBI) in female, but not male, mice. While both sexes exhibited comparable baseline aerobic capacity and similar reductions in maximal oxygen uptake (VO_2_ max) acutely after TBI, only female mice showed significant improvements in exercise tolerance and VO_2_ max following a 10-day treadmill training regimen. Notably, female mice maintained higher voluntary wheel running activity and energy expenditure post-injury and exhibited smaller lesion volumes compared to males. In contrast, injured males displayed persistent exercise intolerance and failed to improve with training, suggesting a sex-specific resistance to aerobic adaptation. These findings highlight a robust sex difference in the recovery of exercise capacity after TBI, with important implications for the design of individualized rehabilitation strategies. There is growing interest in using aerobic exercise as a low-risk, low-cost intervention to improve functional outcomes following TBI (Alarie et al., 2021; Snowden et al., 2023). However, several longstanding clinical and practical barriers have limited the widespread adoption of exercise-based rehabilitation. Individuals with TBI frequently report exercise intolerance, including symptom exacerbation and an inability to sustain exertion (Antonellis et al., 2024). Furthermore, the optimal exercise intensity and duration that maximizes recovery without triggering adverse physiological effects remains undefined, and it is unclear whether exercise should be titrated according to cardiovascular metrics or symptom threshold (Bezherano et al., 2021). Finally, there is a persistent, albeit waning, clinical legacy of recommending prolonged rest or activity restriction in the aftermath of TBI (Weil et al., 2023).

Our current findings, together with our previous work, highlight several key features of the complex interaction among exercise, cardiometabolic function, and TBI recovery. First, aerobic exercise has the potential to reduce neuropathology and improve functional outcomes post-TBI (Karelina et al., 2021; White et al., 2023). Second, we observed marked sex differences in responsiveness to exercise. Female mice demonstrated significant gains in aerobic capacity and metabolic function following treadmill training, including increased time to exhaustion and improved VO_2_ max. In contrast, while males may benefit behaviorally from low-to moderate-intensity exercise (White et al., 2023), they failed to show corresponding improvements in aerobic fitness or endurance following the same training regimen.

The observed alterations across multiple ETC complexes suggest that TBI and aerobic exercise engage distinct, sex-dependent programs of cardiac mitochondrial function. The increase in cardiac complex I and complex III activity in exercised females points to enhanced flux through the NADH-linked oxidative pathway (Lenaz et al., 2006; Nolfi-Donegan et al., 2020), consistent with a shift toward carbohydrate utilization (as supported by elevated RER) and improved oxidative throughput (Atlante and Valenti, 2023; Goedecke et al., 2000). Complex IV activity, which represents the terminal step in the electron transport chain (Nolfi-Donegan et al., 2020), was increased by injury regardless of sex or exercise, suggesting a potential compensatory response to injury-induced mitochondrial stress that may act to maintain electron flux. Interestingly, complex V activity, reflecting ATP synthase function (Nolfi-Donegan et al., 2020), was elevated in females across all conditions, which is consistent with other reports of enhanced mitochondrial efficiency and substrate-stimulated respiration in female rodents (Colom et al., 2007; Hertel Ribeiro et al., 2016; Ventura-Clapier et al., 2017). In contrast, males showed limited evidence of ETC remodeling, with only complex IV activity increasing after exercise in injured animals. This isolated change may reflect an incomplete or qualitatively different adaptation in response to injury. Together, these findings suggest that mitochondrial reprogramming occurs to a greater extent in females acutely following TBI and moderate exercise, potentially contributing to the improved aerobic recovery observed in this group.

Notably, female mice exhibited smaller lesion volumes than males, consistent with prior reports (reviewed in Rubin and Lipton, 2019). Differences in injury severity, therefore, were likely to contribute to the sex difference observed in post-injury outcomes. However, several observations indicate that injury severity alone is unlikely to account for the presented findings. First, lesion volume did not significantly correlate with exercise capacity or metabolic measures, suggesting that the extent of tissue damage was not the primary determinant of functional recovery. Second, our previous work using the same injury parameters demonstrated that male mice exhibited significant behavioral and neuropathological improvement following low-moderate intensity treadmill exercise (Karelina et al., 2021; White et al., 2023). Thus, while females may benefit from smaller lesions, this study’s outcomes are likely the result of a combined influence of injury severity, sex-specific physiology, and behavioral/cardiovascular variables that promote voluntary activity and motivation for sustained exertion.

Accordingly, these findings highlight a sexually dimorphic response to exercise after injury and represent fundamental differences in the physiological interpretation of exercise stimuli following brain injury. Identical training regimens may impose non-equivalent internal loads across sexes. While females appear to operate within an adaptive zone where moderate-intensity training supports both metabolic and neurobiological benefits, males may be constrained by a narrower therapeutic window. Specifically, although higher-intensity exercise may be necessary to drive cardiometabolic adaptation in injured males, our prior work demonstrates that such intensities worsen lesion pathology and impair cognitive recovery (White et al., 2023). As a result, male mice may be under-stimulated by moderate-intensity exercise, yet unable to tolerate the higher intensities required for aerobic gains, resulting in blunted adaptation despite training. This mismatch between the intensity needed for peripheral benefit and the threshold for central injury may represent a core barrier to rehabilitation in males after TBI. Alternatively, the limiting variable for males may be duration, rather than intensity. Many endurance training studies report that some individuals exhibit minimal cardiometabolic benefit, so-called “non-responders”; however, more recent evidence suggests that this may reflect inadequate exercise dose rather than true physiological resistance (Bouchard and Rankinen, 2001; Timmons et al., 2010). For example, among healthy individuals who were randomly assigned to receive 1-5 weekly exercise sessions in a 6-week period, more than 1/4 of individuals that received between 3 or fewer weekly sessions did not exhibit improvements in cardiovascular fitness. When those same non-responsive individuals participated in a second 6-week period with 2 additional exercise sessions per week, non-responsiveness was eliminated in all participants (Montero and Lundby, 2017). Thus, it seems highly likely that male mice could experience gains in cardiovascular fitness even after CCI if the duration and/or intensity of exercise were increased.

Interestingly, we observed no significant difference in baseline VO_2_ max between sham-injured male and female mice, suggesting that aerobic capacity at rest was similar across sexes. However, in a separate cohort of animals housed in metabolic cages, females voluntarily ran substantially greater distances than males, independent of injury. This finding is consistent with prior reports on sex differences in voluntary activity in young mice (Bartling et al., 2017; Janowski et al., 2024; Kim et al., 2023). The dissociation between matched VO_2_ max and dramatically different voluntary running behavior suggests that VO_2_ max alone does not fully capture the physiological or behavioral traits underlying exercise responsiveness. Rather, females may possess greater metabolic efficiency, fatigue resistance, or reward-linked motivation that predispose them to sustained aerobic activity and make them more likely to benefit from structured training (Ansdell et al., 2020). These findings raise the possibility that voluntary running behavior may serve as a proxy for broader physiological traits that modulate aerobic adaptability, particularly in the context of injury and recovery.

## Conclusion

From a clinical standpoint, these findings have both theoretical and practical significance. Exercise is known to confer cardiovascular, metabolic, affective, and neurovascular improvements, and has been increasingly recognized as a potential tool to promote recovery after brain injury (Huang and Wong, 2025; Nystoriak and Bhatnagar, 2018; Wender et al., 2024; Zhang and Gao, 2021). Yet, emerging evidence suggests that exercise may be detrimental if initiated too early, performed too intensely, or sustained for too long, particularly in the acute post-injury period (Griesbach, 2011; Griesbach et al., 2012; Piao et al., 2013). Our prior experimental work demonstrated that high-intensity aerobic training worsens both cognitive and neuropathological outcomes in male mice (White et al., 2023), underscoring the need for carefully titrated approaches to developing exercise regimens after TBI.

The present study directly addresses the key gaps outlined in the introduction. First, we show that injury itself alters voluntary activity and whole-body metabolism in a sex-dependent manner, with injured females maintaining higher spontaneous activity and distinct metabolic profiles compared to males. Second, we demonstrate that moderate-intensity aerobic training partially restores exercise tolerance and cardiorespiratory fitness after TBI in female mice, indicating that males may require different training parameters to achieve comparable adaptations. Finally, we identify sex-specific cardiac mitochondrial responses, such that aerobic training enhanced ETC activity to a greater extent in females. Taken together, these findings highlight sex as a fundamental biological variable shaping response to exercise after TBI.

A key takeaway is that it is essential to consider what exercise is intended to accomplish, as different therapeutic goals may lead to divergent clinical recommendations. This underscores a critical distinction: while physical deconditioning is undesirable, it can often be reversed during later recovery (Hamel and Smoliga, 2019). Neurological injury, however, may be less reversible, particularly if exacerbated by premature or excessive exertion (Kreber and Griesbach, 2016). In this context, exercise prescriptions during early recovery may need to prioritize neurological tolerance over cardiovascular targets. This perspective aligns with symptom-based clinical approaches that titrate training to ∼80% of the exertional threshold where symptoms emerge, a method that has shown success in reducing persistent post-concussive symptoms (Bezherano et al., 2021; Leddy et al., 2019; Mercier et al., 2020). Conversely, exercise prescriptions based solely on age, fitness, or heart rate, without accounting for sex or injury severity, risk imposing suboptimal or even harmful regimens. Future studies should aim to disentangle the physiological mechanisms that underlie sex-specific exercise responsiveness following TBI. Titrating exercise paradigms to physiological responses (e.g., lactate threshold, heart rate variability) rather than externally defined workloads may offer more precise, individualized rehabilitation strategies. Ultimately, understanding how these variables interact with sex and injury severity could significantly improve post-TBI recovery outcomes.

## Data Availability

The raw data supporting the conclusions of this article will be made available by the authors, without undue reservation.
