# Supplementary material for: Aerobic training improves exercise capacity after traumatic brain injury in female, but not male, mice
Source: Front Physiol. 2025 Oct 30;16:1700462. doi: 10.3389/fphys.2025.1700462 (PMC12611811; doi:10.3389/fphys.2025.1700462)
Supplement: Supplementary file 1 [file Supplementaryfile1.docx]

Supplemental Figure 1. Cumulative food intake. Food intake was significantly greater in male mice but did not differ significantly by injury status (p = 0.08 in females). * indicates significant sex difference, p < 0.05.
